# Coronary artery spasm due to acute myocarditis in an adolescent: a case report

**DOI:** 10.1186/s12887-022-03354-7

**Published:** 2022-05-24

**Authors:** Hoon Ko, Taehong Kim, Hyoung Doo Lee, Joung-Hee Byun, Ki Seok Choo

**Affiliations:** 1 Department of Pediatrics, Pusan National University Yangsan Hospital, 20, Geumo-ro, Mulgeum-eup, Yangsan-si, Gyeongsangnam-do 50612 South Korea; 2 Department of Radiology, Pusan National University Yangsan Hospital, Yangsan-si, 50612 South Korea

**Keywords:** Acute myocarditis, Coronary artery spasm, ST-segment elevation, Case report

## Abstract

**Background:**

Myocarditis refers to the inflammation of the myocardium caused by infection or autoimmune disease that may or may not present with clinical manifestations, such as gastrointestinal symptoms, dyspnea, chest pain, or sudden death. Although myocarditis and coronary artery vasospasm may mimic ST-segment elevation myocardial infarction (STEMI) with normal coronary arteries on angiography, acute myocarditis rarely causes coronary artery spasm. Here, we report a case of coronary artery spasm with reversible electrocardiographic changes mimicking STEMI in an adolescent with acute myocarditis.

**Case presentation:**

A 15-year-old boy present with sudden-onset repeated chest pain following a 3-day history of flu-like illness. Cardiac biomarkers were significantly elevated. Electrocardiography showed ST-segment elevation in the absence of detectable vasospasm on coronary angiography. These findings were consistent with the diagnosis of coronary artery spasm secondary to acute myocarditis. Treatment with immunoglobulin for 2 days improved his condition. The patient was discharged on the 12th day with complete resolution of symptoms and normalization of electrocardiogram findings.

**Conclusions:**

We reported a case of coronary artery spasm due to acute myocarditis. This study highlights the importance of considering coronary artery spasm due to acute myocarditis as a differential diagnosis in patients presenting with signs of STEMI as these diseases have different medical management strategies.

## Background

Myocarditis refers to the inflammation of the myocardium diagnosed using established histological, immunological, and immunohistochemical criteria [1]. Cell death and injury due to inflammation may cause structural and functional cardiac abnormalities presenting with various clinical manifestations. Because acute myocarditis may mimic ST-segment elevation myocardial infarction (STEMI), its early identification may have important therapeutic and prognostic implications [2, 3]. Coronary artery spasm is a sudden, intense, transient and reversible vasoconstriction of an epicardial coronary artery that can also result in ST-segment elevation. However, acute myocarditis rarely causes coronary artery spasm.

Here, we report an unusual case of coronary artery spasm secondary to acute myocarditis in a 15-year-old boy presenting with chest pain, ST-segment elevation on electrocardiogram (ECG), and highly elevated cardiac biomarkers in the absence of detectable vasospasm on coronary angiography.

## Case presentaion

A 15-year-old boy presented with sudden-onset chest pain at 02:30 AM characterized as squeezing on the left parasternal area that persisted for over 2 hours following a 3-day history of flu-like illness. Although this resolved spontaneously at first, he experienced chest pain and dyspnea 18 hours later, which prompted a visit to the emergency room. During admission, his creatine kinase MB fraction was elevated at 49.2 ng/ml (normal range: 0 to 3.6 ng/ml; AMI cut off: 5.0 ng/ml) and Troponin-I was elevated at 15.5 ng/ml (normal range: 0 to 0.056 ng/ml; AMI cut off: 0.6 ~ 1.5 ng/ml). ECG showed ST-segment elevation at I, aVL, and precordial leads.

The patient was then transferred to the emergency room of our hospital. He was middle school student with no known comorbidities, such as hypertension, diabetes, or cardiovascular disease. He had no family history of coronary artery disease. He denied alcohol consumption and smoking.

His blood pressure was 110/70 mmHg, the heart rate was 86 bpm, the respiratory rate was 15/min, and the body temperature was 38.5 °C. His oxygen saturation at room air was 99%.

**Fig. 1:**
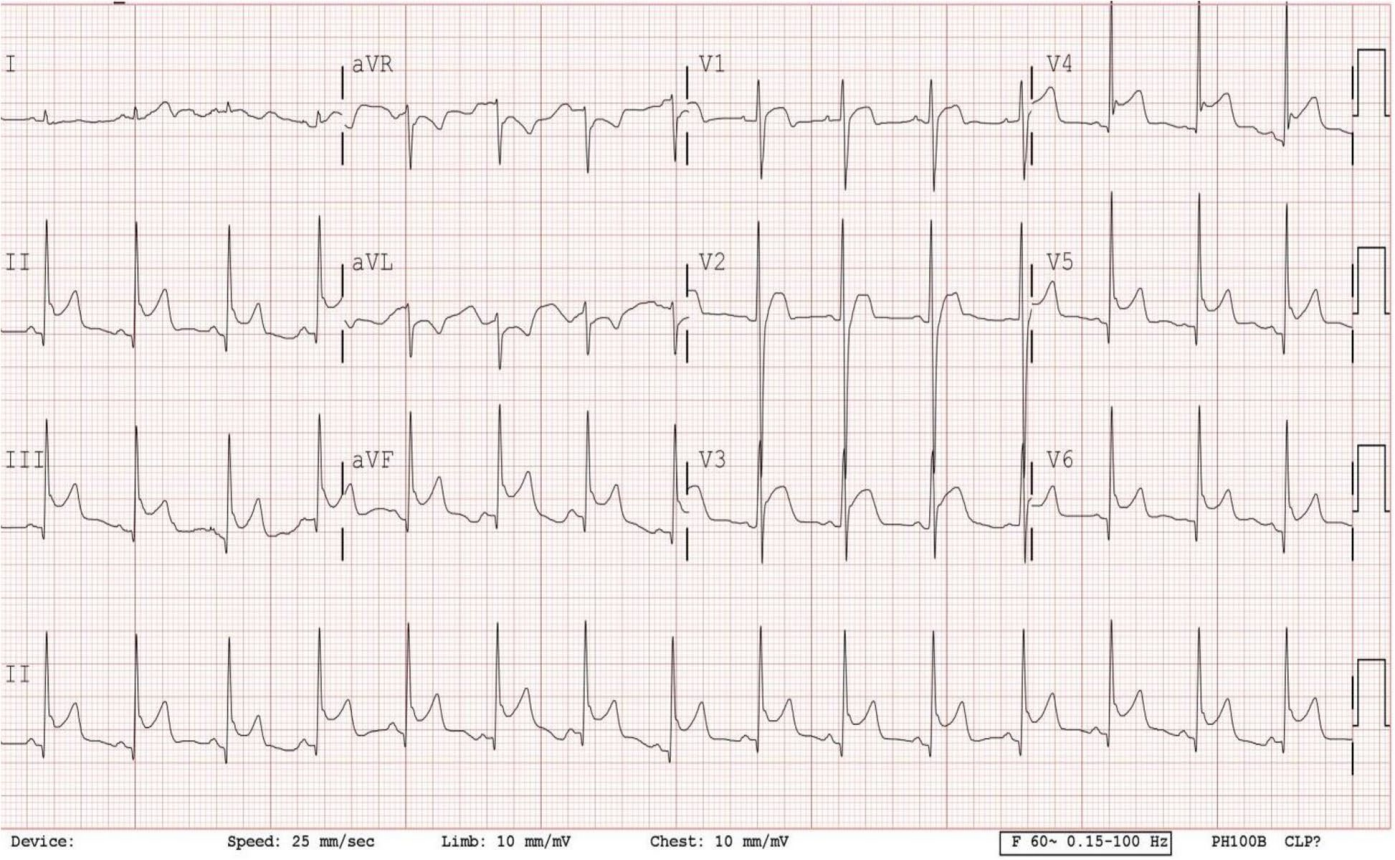
12-lead electrocardiogram showed sinus rhythm and ST-segment elevation in inferior lead (II, III, aVF), and precordial leads, accompanied by ST-segment depression in in aVL at the time of admission

Physical examination, including cardiovascular examination, was unremarkable. The chest X-ray revealed normal heart size and clear lung fields. ECG showed sinus rhythm with ST-segment elevation in the inferior and precordial leads (Fig. 1). Transthoracic echocardiography revealed normal left ventricular ejection fraction (66.04%) and very mild anteroseptal hypokinesis.

**Fig. 2:**
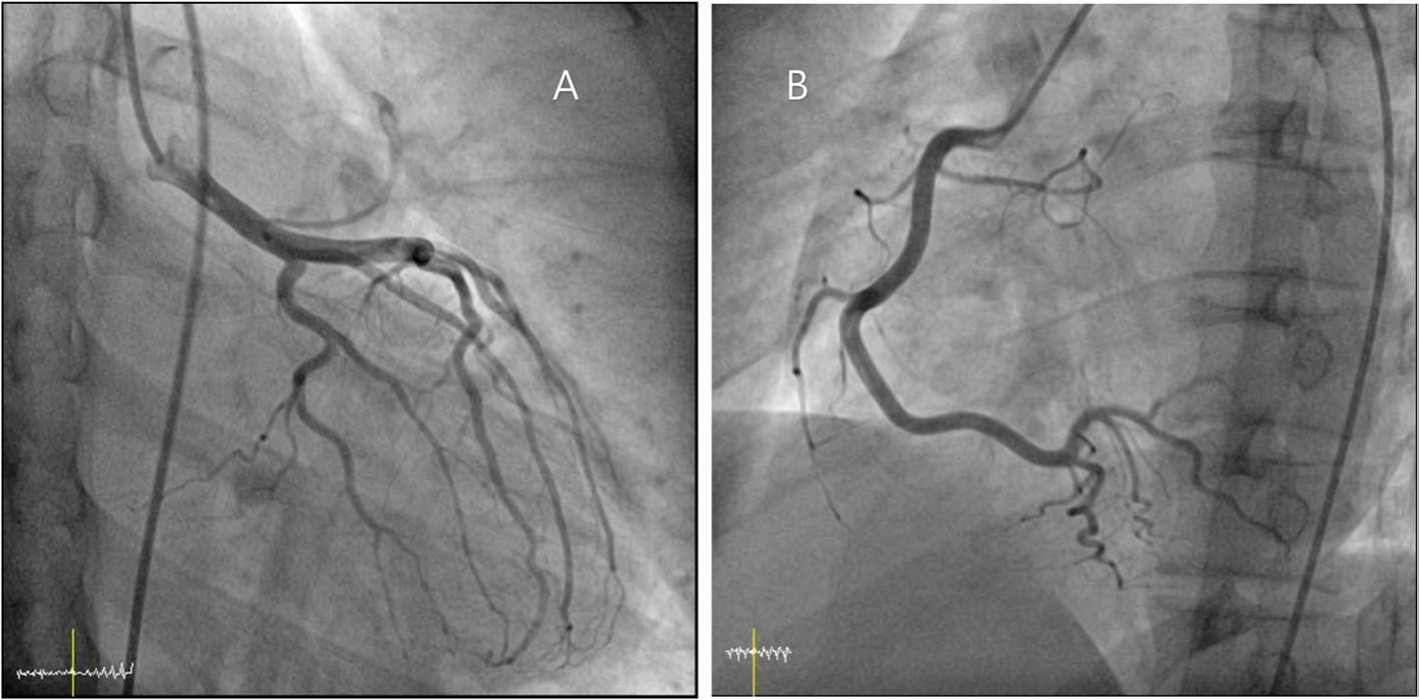
Coronary angiography demonstrated normal left (**A**) and right (**B**) coronary arteries

**Fig. 3:**
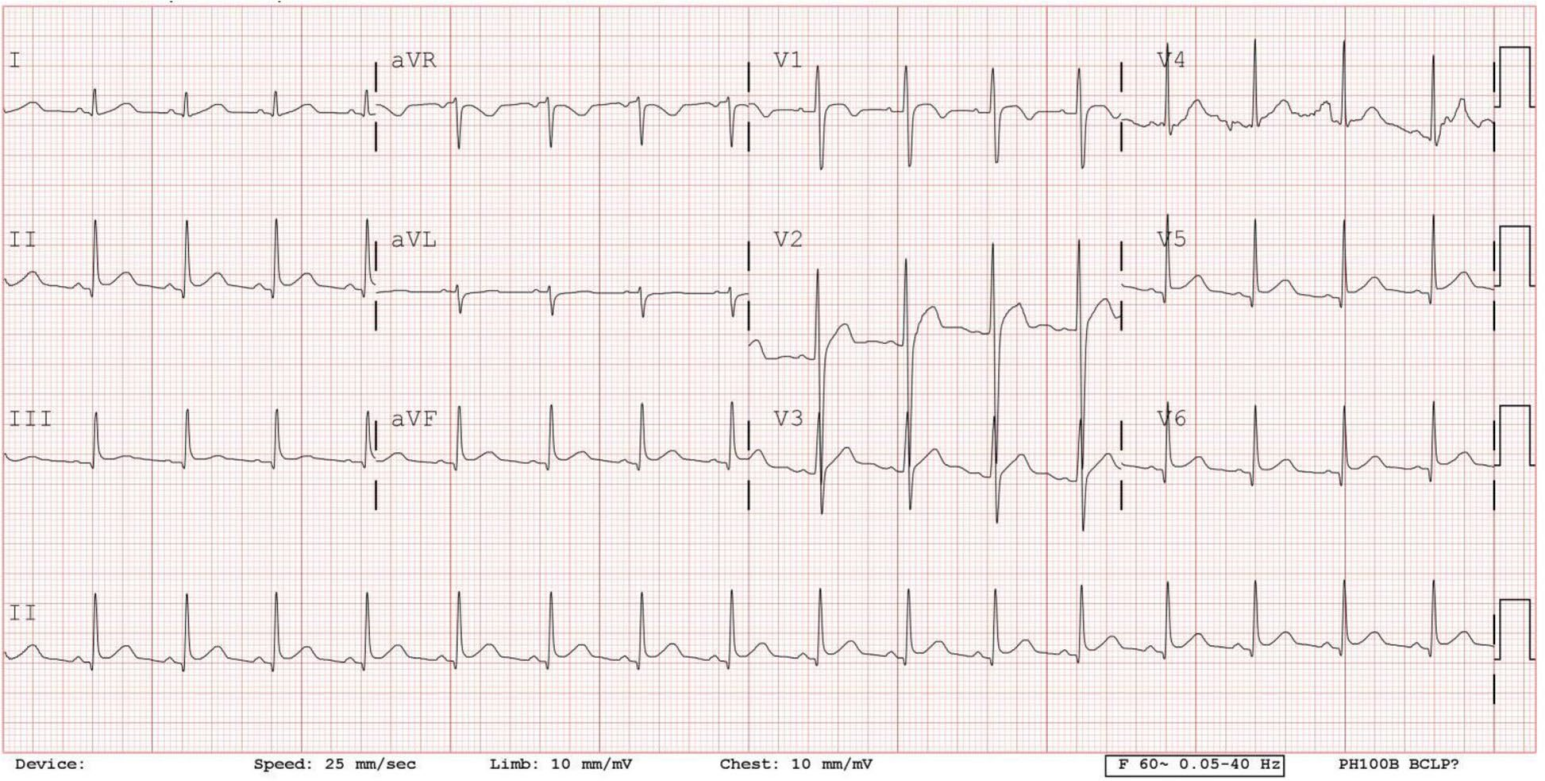
12-lead electrocardiogram on 4th day of admission showing sinus rhythm with mild ST segment change in II, V5 and V6

After 23 h from the onset of chest pain, myoglobin was elevated at 363.2 ng/mL (normal range: 12 to 80 ng/ml), high sensitive-Troponin I increased to 21,538.68 pg/mL (normal range: 0 to 19.8 pg/ml), and creatine kinase MB fraction also increased to 170.0 ng/mL (normal range: 0.5 to 3.1 ng/ml). To differentiate STEMI or other combined coronary vessel anomalies, coronary angiography was performed, and it revealed normal coronary arteries (Fig. 2). Treatment with immunoglobulin for 2 days improved his condition and cardiac markers (myoglobin: 8.6 ng/mL, high sensitive-Troponin I: 7466 ng/mL, creatine kinase MB fraction: 3.0 ng/ml) and ECG. On the 3rd night of admission, he experienced another episode of chest pain during sleeping with increased cardiac markers (myoglobin: 10.5 ng/ml, high sensitive-Troponin I: 16,164.61 ng/ml, creatine kinase MB fraction: 7.8 ng/ml) and ST-segment change on ECG (Fig. 3). The chest pain resolved without medication.

**Fig. 4:**
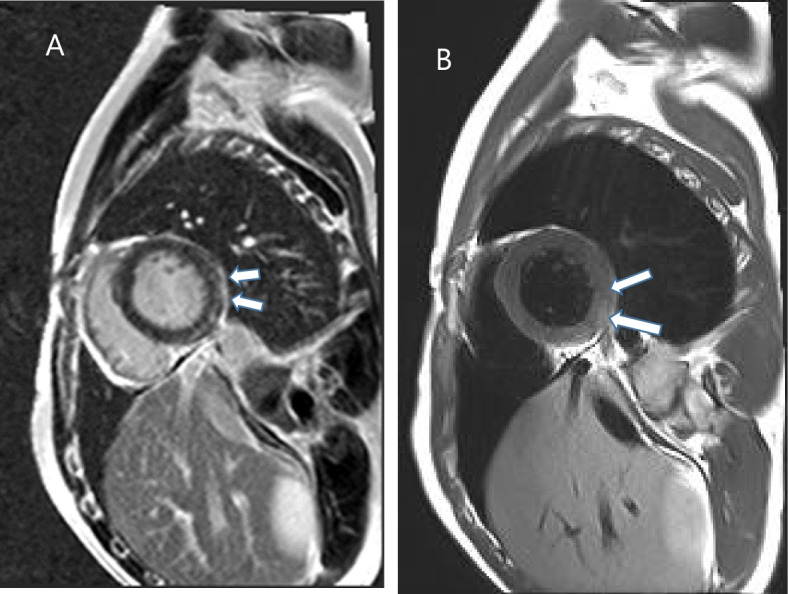
Cardiac magnetic resonance image. Short-axis late gadolinium enhanced (LGE) MR images (**a**) show subepicardial enhancement (arrow) in the anterolateral and inferolateral wall of mid base left ventricle (LV) level. Short-axis T2-weighted MR images (**b**) show high signal intensities (arrows) in those regions with enhancement regions, suggesting myocardial edema

**Fig. 5:**
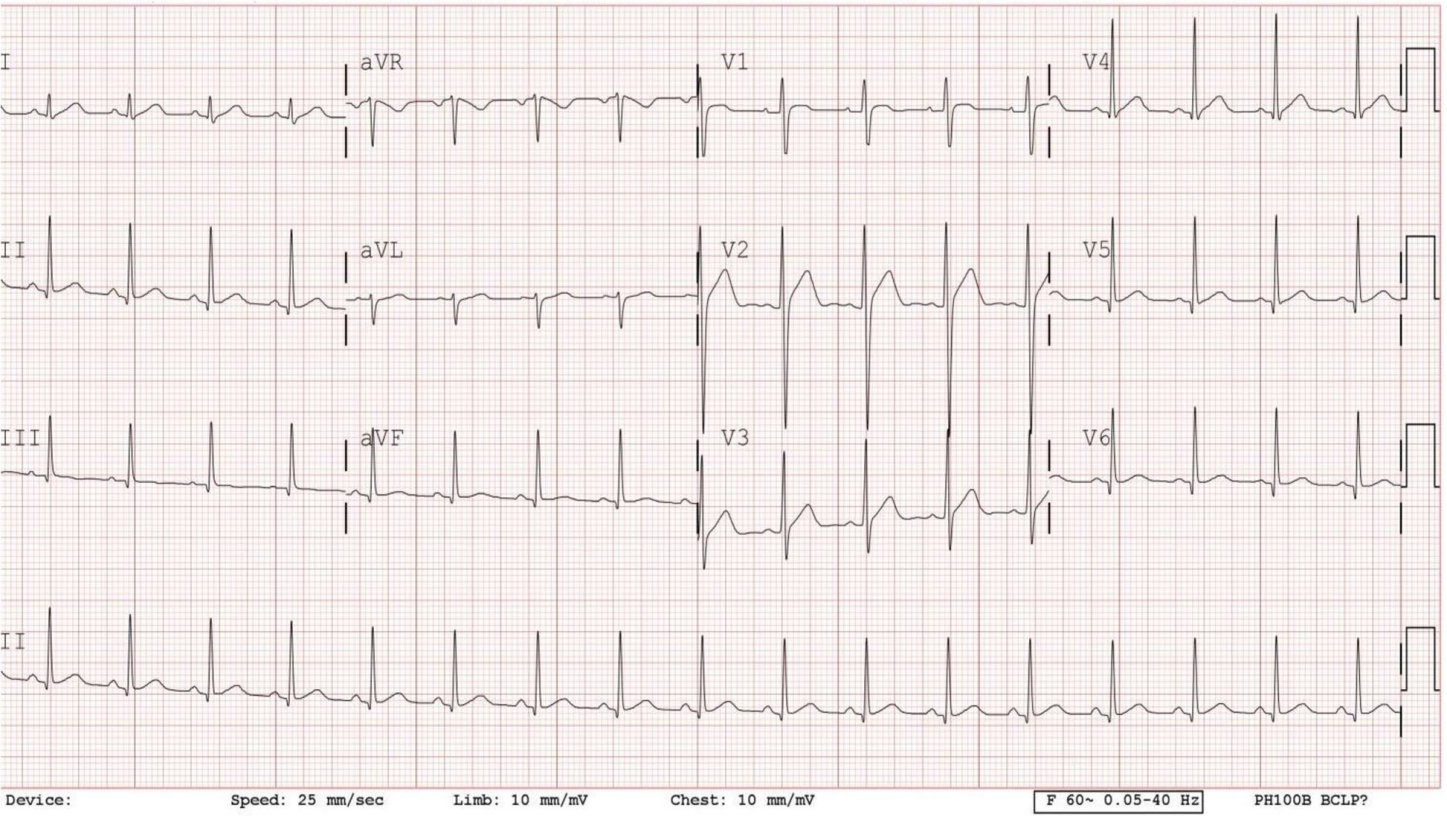
Repeat electrocardiogram on the day 12 of hospitalization showing near complete resolution of ST-segment elevation

All of these findings were consistent with the presumptive diagnosis of acute myocarditis and coronary artery spasm leading to transient ST-segment elevation on ECG. To confirm the diagnosis of myocarditis, we performed cardiac magnetic resonance imaging (MRI) and it showed myocardial edema and subepicardial enhancement in the anterolateral and infero-lateral wall of mid base left ventricle (Fig. 4). On the 12th day of admission, the patient was discharged with complete resolution of symptoms and normalization of the ECG findings (Fig. 5).

## Discussion and conclusions

Myocarditis refers to the inflammation of the myocardium due to infectious (typically viruses) and non-infectious causes (i.e., toxins, hypersensitivity reactions, and autoimmune disease) [4]. The most common causes of viral myocarditis include parvovirus B19, human herpesvirus 6, enterovirus, cytomegalovirus, adenovirus [5].

The pathogenesis of viral myocarditis involves three chronologically and pathologically distinct phases. In the first phase of myocarditis, the virus enters and proliferates in the myocardium, causing direct myocellular necrosis and apoptosis. This is followed by the initiation of the innate immune response. Both the direct myocardial damage cause by the virus and the subsequent immune response result in the cardiomyocytes destruction, mimicking signs and symptoms of acute myocardial infarction, such as focal ST-segment elevation on ECG and elevated serum cardiac enzymes [6]. Following the first phase, patients may recover or progress into the next phase. In the second phase, the adaptive immune response is activated. In the third phase, extensive myocardial injury and dilated cardiomyopathy may develop [5].

Myocarditis may or may not present with several non-specific clinical manifestations, such as gastrointestinal symptoms, dyspnea, chest pain, cardiogenic shock or sudden death. Gastrointestinal symptoms (e.g., abdominal pain, nausea, poor appetite and vomiting) are the most common presentations of acute myocarditis [7]. ECG, cardiac biomarkers (i.e., creatinine kinase MB fraction and troponin I), cardiac MRI and endomyocardial biopsy are useful tools in the diagnosis of acute myocarditis [5, 6].

Prinzmetal et al. postulated the concept of coronary artery spasm by describing pain associated with transient non-progressive ST-segment elevation appeared at rest or during regular activity but was not brought on by exercise or emotional disturbance. Coronary artery spasm is a reversible vasoconstriction due to a spontaneous vascular smooth muscle hypercontractility and vascular wall hypertonicity. This cause luminal narrowing of normal or atherosclerotic coronary arteries, compromising the myocardial blood flow and usually leads to transient myocardial ischemia, myocardial infarction, and sudden death [8, 9]. A typical clinical presentation of coronary vasospasm is variant angina, in which chest pain usually occur at rest between night and early morning, not induced by exercise or effort during daytime and spasms are usually brief, but can persist for more than 15 minutes. This is an important feature of the history and often used in diagnostic criteria [10]. Although the exact pathophysiological mechanism of coronary artery spasm is not clearly understood, abnormal response of the autonomic nervous system, endothelial dysfunction, inflammation, oxidative stress, vascular smooth muscles hypercontractility, and other factors (e.g. magnesium deficiency, inheritance, or specific anatomy of the coronary artery) have been proposed [11].

Myocarditis and coronary artery spasm are well-known causes of ST-segment elevation, mimicking acute myocardial infarction. The cardiac MRI findings, past history, age, risk factors, subjective symptoms, value of cardiac markers, dynamic changes in ECG are very useful for differential diagnosis. To the best of our knowledge, coronary artery spasm precipitated by acute myocarditis has rarely been reported in the pediatric population. The pathophysiological mechanism of coronary artery spasm due to acute myocarditis remains unclear. There are several mechanisms suggested that vasoactive substances (e.g. thromboxane A2), myocarditis-induced endothelial dysfunction, smooth muscle hyperactivity may induce coronary artery spasm in acute myocarditis patients [12–14].

In our case, myocarditis was confirmed using cardiac MRI. Moreover, the coronary artery spasm was consistent with the features of chest pain, ST-segmental elevation and spontaneous resolution, and normal coronary angiographic findings. We presumed that acute myocarditis precipitated coronary artery spam as a result of myocarditis-induced coronary endothelial dysfunction or coronary smooth cell hyperactivity similar to the findings of other studies [12–14].

In conclusion, we reported a case of coronary artery spasm due to acute myocarditis. This study highlights the importance of considering coronary artery spasm due to acute myocarditis as a differential diagnosis in patients presenting with signs of STEMI as these diseases have different medical management strategies.

## Data Availability

The data presented in this study are available on reasonable request from the corresponding author.

## Abbreviations

STEMI: ST-segment elevation myocardial infarction
ECG: Electrocardiography
MRI: Magnetic resonance imaging

## References

[1] Richardson P, McKenna RW, Bristow M, Maisch B, Mautner B, O’Connell J (1996). Report of the 1995 world health organization/international society and federation of cardiology task force on the definition and classification of cardiomyopathies. Circulation.

[2] Karjalainen J, Heikkila J (1999). Incidence of three presentation of acute myocarditis in young men in military service. A 20-year experience. Eur Heart J.

[3] Mottard N, Mewton N, Bonnefoy E, Abdellaoui M, Revel D, Kirkorian G (2008). Acute myocarditis mimicking lateral myocardial infarction. Anaesth Intensive Care.

[4] Kindermann I, Barth C, Mahfound F, Ukena C, Lenski M, Yilmaz A (2012). Update on myocarditis. J Am Coll Cardiol.

[5] Singh RK, Yeh JC, Price JF (2016). Diagnosis and treatment strategies for children with myocarditis. Prog Pediatr Cardiol.

[6] Pollack A, Kontorovich AR, Fuster V, Dec GW (2015). Viral myocarditis- diagnosis, treatment options and current controversies. Nat Rev Cardiol.

[7] Butts RJ, Boyle GJ, Deshpande SR, Gambetta K, Knecht KR, Prada-Ruiz CA (2017). Characteristics of clinically diagnosed pediatric myocarditis in a contemporary multi-center cohort. Pediatr Cardiol.

[8] Pasupathy S, Tavella R, Beltrame JF (2017). Myocardial infarction with nonobstructive coronary arteries (MINOCA): the past, present, and future management. Circulation.

[9] Ford TJ, Rocchiccioli P, Good R, McEntegart M, Eteiba H, Watkins S (2018). Systemic microvascular dysfunction in microvascular and vasospastic angina. Eur Heart J.

[10] JCS joint working group (2014). Guidelies for diagnosis and treatment of patients with vasospastic angina (coronary spastic angina) (JCS 2013). Circ J.

[11] Teragawa H, Oshita C, Ueda T (2018). Coronary spasm: it’s common, but it’s still unsolved. World J Cardiol.

[12] Silva D, Marques P, Martins S (2010). Coronary artery vasospasm and acute myocarditis: a rare association. Rev Port Cardiol.

[13] Yilmaz A, Mahrholdt H, Athanasiadis A, Vogelsberg H, Meinhardt G, Voehringer M (2008). Coronary vasospasm as the underlying cause for chest pain in patients with PVB-19 myocarditis. Heart.

[14] Iwasaki K, Kusachi S, Tominaga Y, Kita T, Taniguchi G (1991). Coronary artery spasm demonstrated by coronary angiography in a patient with acute myocarditis resembling acute myocardial infarction: a case report. Jpn J Med.

